# Screening Methods for Diagnosing Cystic Fibrosis-Related Diabetes: A Network Meta-Analysis of Diagnostic Accuracy Studies

**DOI:** 10.3390/biom11040520

**Published:** 2021-03-31

**Authors:** Vera Dóra Izsák, Alexandra Soós, Zsolt Szakács, Péter Hegyi, Márk Félix Juhász, Orsolya Varannai, Ágnes Rita Martonosi, Mária Földi, Alexandra Kozma, Zsolt Vajda, James AM Shaw, Andrea Párniczky

**Affiliations:** 1 Institute for Translational Medicine, Medical School, University of Pécs, 7624 Pécs, Hungary; arev44@gmail.com (V.D.I.); soos.alexandra5622@gmail.com (A.S.); szaki92@gmail.com (Z.S.); hegyi2009@gmail.com (P.H.); flixjuhsz@gmail.com (M.F.J.); varannaiorsolya@gmail.com (O.V.); agirmartonosi@gmail.com (Á.R.M.); foldimarcsi4@gmail.com (M.F.); kozmaa0219@gmail.com (A.K.); 2 Heim Pál National Pediatrics Institute, 1089 Budapest, Hungary; heimpalendo@gmail.com; 3 Doctoral School of Clinical Medicine, University of Szeged, 6720 Szeged, Hungary; 4 János Szentágothai Research Center, University of Pécs, 7624 Pécs, Hungary; 5 Translational and Clinical Research Institute, Newcastle University, Newcastle upon Tyne NE1 7RU, UK; jim.shaw@ncl.ac.uk

**Keywords:** cystic fibrosis, cystic fibrosis-related diabetes, continuous glucose monitor, oral glucose tolerance test

## Abstract

Background: Cystic fibrosis-related diabetes (CFRD) has become more common due to higher life expectancy with cystic fibrosis. Early recognition and prompt treatment of CFRD leads to improved outcomes. Methods: We performed a network meta-analysis (NMA) in order to identify the most valuable diagnostic metrics for diagnosing CFRD out of available screening tools (index test), using the oral glucose tolerance test as a reference standard. Pooled sensitivity (Se), specificity (Sp), and superiority indices were calculated and used to rank the index tests. Results: A total of 31 articles with 25 index tests were eligible for inclusion. Two-day, continuous glucose monitoring (CGM) ranked the highest (Se: 86% Sp: 76%), followed by glucose measurement from blood capillary samples (Se: 70%, Sp: 82%) and three-day CGM (Se: 96%, Sp: 56%). When we compared the CGM of different durations, two-day CGM performed best (Se: 88%, Sp: 80%), followed by three-day (Se: 96%, Sp: 59%) and six-day CGM (Se: 66%, Sp: 79%). Conclusions: Considering its overall performance ranking, as well as the high sensitivity, two-day CGM appears to be a promising screening test for CFRD.

## 1. Introduction

Cystic fibrosis (CF) is an autosomal recessive genetic disease caused by mutations of the cystic fibrosis transmembrane conductance regulator (CFTR) gene. The pathogenic mutations cause abnormal chloride transport across secretory epithelial cells, leading to thick, sticky mucus production, mainly affecting the lungs and the digestive system [1]. Due to advances in treatment and diagnostics, the life expectancy of CF patients has increased over the last decade. Consequently, previously rare extra-pulmonary complications are now highly prevalent. Risk factors for developing cystic fibrosis-related diabetes (CFRD) include pancreatic insufficiency, severe genotype (deltaF508 homozygotes), and increased age; consequently it affects approximately 20% of adolescent and 40–50% of adult individuals with CF [2,3]. CFRD is categorized as a specific subtype of diabetes mellitus (DM) in the 2018 American Diabetes Association (ADA) guidelines, sharing features with both type 1 and type 2 diabetes [4]. In the initial stage of the disease, insulin and glucagon deficiency develop due to pancreatic islet cell dysfunction and loss in combination with exocrine pancreatic insufficiency. In addition, acute and chronic inflammation result in fluctuating insulin resistance, which may play a significant role in the pathogenesis. CFRD is associated with diminished lung function, suboptimal nutritional status, and increased mortality [1,5]. Despite the resemblances to other types of diabetes, pathophysiology of CFRD is a fundamentally different, and it should be considered independently regarding diagnosis and treatment.

The current gold-standard for CFRD screening is the 2 h oral glucose tolerance test (OGTT) (1.75 g glucose/kg body weight, maximum = 75 g). It is recommended that this is performed annually in all people with CF beginning at 10 years age and with no previous diagnosis of CFRD. [4] The CFRD diagnostic criteria, according to the ADA guidelines for stable CF, are defined as 2 h OGTT (T120′) plasma glucose ≥ 11.1 mmol/L, fasting plasma glucose (FPG) ≥ 7.0 mmol/L, haemoglobin A1c (HbA1C) ≥ 48 mmol/mol (6.5%), or classical symptoms of diabetes (polyuria and polydipsia) in the presence of a random glucose level ≥ 11.1 mmol/L [6].

The current gold standard procedure is inconvenient, due to fasting and being time-consuming, and does not represent an everyday glucose homeostasis [4,7]. Therefore, we cannot leave out of consideration the fact that OGTT T120′ plasma glucose can be normal even if other screening methods, such as continuous glucose monitoring (CGM), reveal abnormal daily glucose excursions. Management with insulin and strict glycaemic control are accompanied by improved clinical outcomes and increased life expectancy. Early recognition of glucose homeostasis abnormalities is thus clearly important and an area of active research [8].

We undertook a network meta-analysis (NMA) to compare the diagnostic performance of the currently available screening tools for the diagnosis of CFRD, and to identify a potential alternative screening tool to the formal OGTT in CF patients. NMA structure allows to simultaneously compare multiple diagnostic tests at multiple thresholds to the gold standard at the same time.

## 2. Materials and Methods

### 2.1. Protocol and Registration

The network meta-analysis of multiple diagnostic tests (NMA-DT) is reported according to the Preferred Reporting Items for Systematic Review and Meta-Analysis (PRISMA) for Network Meta-Analyses Statement [9]. The protocol of the meta-analysis was registered on the International Prospective Register of Systematic Reviews (PROSPERO) under the registration number CRD42020160389.

### 2.2. Data Sources and Search Strategy

We conducted a systematic literature search using five medical databases, including MEDLINE (via PubMed), EMBASE, Web of Science Core Collection, Scopus, and CENTRAL in October 2019 (our search strategy is detailed in Table S1). Additionally, we performed a manual search for cited and citing reports of the eligible articles, revised through Google Scholar, applying the same eligibility criteria as for the database search.

### 2.3. Eligibility Criteria

The inclusion criteria for the study were the following: (1) prospective diagnostic accuracy studies, in which patients were previously diagnosed with CF; (2) studies must use OGTT T120′ value as the reference standard; and (3) studies must evaluate one or more diagnostic methods, comparing to the reference standard, as index tests. Studies were not limited based on the age of enrolled participants—both pediatric and adult studies were considered eligible.

Exclusion criteria were the following: (1) records examining only CF patients with previously known impaired glucose tolerance (IGT) or diabetes; (2) studies that assessed FPG (T0′) or 1-h OGTT (T60′) values as the only index test; and (3) fewer than five participants.

We included both full-text papers and conference abstracts to reduce publication bias. If useful data were available for an incomplete proportion of the study population, we used only the valid data from the smaller number of participants. In case of conspicuous overlapping populations between studies (matching authors, hospitals, index tests, and same study periods), we selected the most recent full text article rather than conference abstract and larger over smaller sample size. Although we included studies with potential risk of overlapping study populations (same authors, same hospitals, same or partially same study period but different index tests), we created two network analyses to avoid any over-representation of data. One network included all studies regardless of potential overlapping populations, and in the second, overlaps were avoided by evaluating the period and place of enrollment, study authors, eligibility criteria, and baseline characteristics of participants. We chose studies with CGM over other index tests, full-text articles over conference abstracts, and greater sample size over small sample size.

### 2.4. Selection and Data Collection Process

Two independent review authors (V.D.I., Á.R.M.) completed all steps of selection and data collection (onto the pre-defined data collection sheet) in duplicate. A third party (Z.S.) resolved any disagreement between the authors. We imported records from each database into EndNote X9 citation manager (Clarivate Analytics, Philadelphia, PA, United States). First, we removed duplicates using the citation manager, and then manually. Remaining records were assessed for inclusion according to the eligibility criteria, by their titles, abstracts, and then by full texts.

All data, according to study type, author, and publication information, demographic data, study period, details of diagnostic methods, number of CFRD participants, number of non-CFRD participants detected by reference standard, and index tests, were collected in the study data table.

### 2.5. Risk of Bias and Applicability

The quality of all included studies was assessed by two independent researchers (V.D.I., Á.R.M.), according to the Quality Assessment of Diagnostic Accuracy Studies-2 (QUADAS-2) tool [10]. Any disagreement was resolved by a third independent person (Z.S.).

### 2.6. Statistical Analysis

After data collection, we created 2 × 2 contingency tables with true positive (TP), true negative (TN), false positive (FP), and false-negative (FN) values for each comparison. If more than one cut-off value was reported for the same index test, we chose the best-performing cut-off. In trials, if the best cut-off was not given, we chose the one corresponding to the value from the currently available CFRD guidelines [4,5,6]. If there was no recommendation in the guidelines, we calculated combined sensitivity and specificity and chose the cut-off with the highest values.

### 2.7. Network Meta-Anaylsis

We performed a network meta-analysis for diagnostic tests (NMA-DT) to investigate which diagnostic method performs best for CFRD diagnosis. NMA-DT allows us to simultaneously compare multiple diagnostic tests at multiple thresholds to the gold standard at the same time. This approach allowed us to make direct (head-to-head) as well as indirect comparisons, given the common comparator (OGTT 120′) in all studies [11]. We considered the evaluation of diagnostic odds ratios (DORs); however, due to continuity correction, results were uninterpretable. DOR is not defined when TP values are zero, which occurred in half of our articles. To assess the relative performance of a diagnostic test, we calculated pooled sensitivity (Se) and specificity (Sp) of the index tests compared to OGTT for the diagnosis CFRD, and ranked them based on the superiority index (SI). The greater the SI, the more accurately a screening test is expected to identify the target condition. “This approach gives more weight to a diagnostic test doing comparatively well on both measures and less emphasis on tests doing relatively poorly on both measures or even doing extremely well on one measure but performing poorly on the other measure” [12].

We illustrated the network graph using STATA (version 15.1). To display the network, we constructed a graph where nodes represent different screening methods, and lines represent head-to-head comparisons. In the network graph, the direct comparisons are presented with edges, the thickness of the edges represents the number of the head-to-head trials, and the size of the nodes correlates with the number of studies [9]. All statistical calculations were performed by R programming language using an ANOVA arm-based model by Nyaga et al. [13].

## 3. Results

### 3.1. Characteristics of the Studies Included

**Figure 1:**
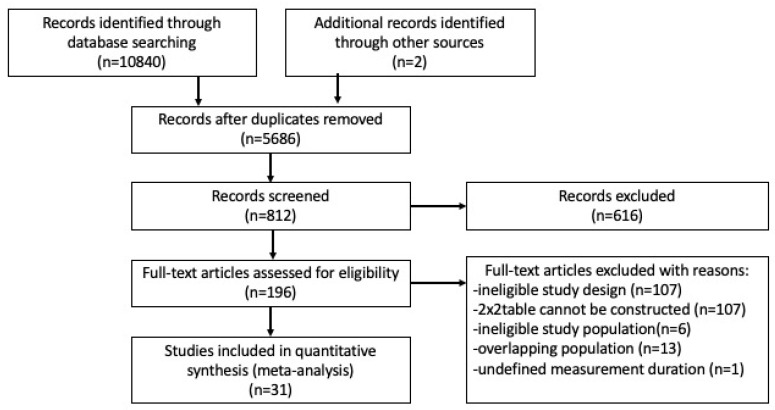
Preferred Reporting Items for Systematic Review and Meta-Analysis (PRISMA) flowchart showing the literature search and study selection process, finding 31 relevant studies. The two articles labeled as “identified through other sources” were located by manually evaluating articles citing and cited by studies included in our review. Study and participant eligibility was applied as defined in the methods section of the article.

We included 31 studies (26 full-text papers and 5 conference abstracts) in the network meta-analysis ([14,15,16,17,18,19,20,21,22,23,24,25,26,27,28,29,30,31,32,33,34,35,36,37,38,39,40,41,42,43,44]). The selection process is shown in Figure 1. Included studies reported on a total of 1976 people with CF, 243 (12.3%) of whom had CFRD. One study included CF patients after lung transplantation without DM [42]. Although we excluded studies including participants with previously identified IGT, we included one study that compared HbA1c to OGTT T120′. It selected patients with clinical decline or reactive symptomatic hypoglycaemia during OGTT, some of whom had impaired or indeterminate glucose tolerance (INDET). INDET means fasting and 2 h glucose levels are normal, but the 1 h glucose level is greater than 11.1 mmol/L.

The studies included a total of 24 index tests, with HbA1c being the most widely used (20 studies). Nineteen studies used a single index test, 10 included two index tests, while 3, 5, and 8 index tests were used in other studies (one study each). Table 1 summarizes the characteristics of the included studies.

### 3.2. Diagnostic Performance of the Index Tests

**Figure 2:**
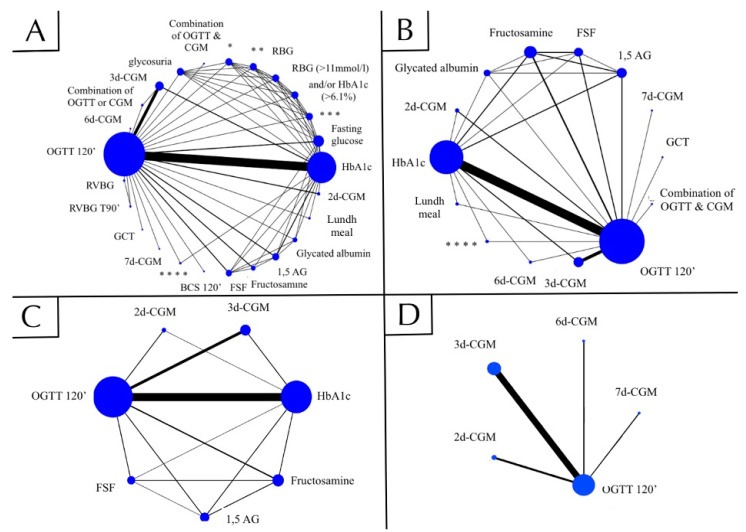
Network graphs. Network graphs show comparisons between examined diagnostic tests and OGTT T120′, used as reference standard. In the network graph, direct comparisons are shown with lines, and the thickness of the lines represents the number of the head-to-head trials, and the size of the nodes correlates with the number of studies. (**A**) Network A summarizes all included studies (*n* = 31), comparing 24 different index tests to the reference standard. (**B**) Network B assesses studies after excluding those, which raises potential risk for overlapping populations. In this network, comprising 25 studies, 13 index tests were compared to the reference standard. (**C**) Network C represents studies (*n* = 23) that analysed index tests (*n* = 6), which had been used in at least two different articles from the study pool of Figure 2B. (**D**) Network D represents studies evaluating different durations of CGM, and included articles (*n* = 10) from the Figure 2B study pool.Abbreviations: d = days; CGM = continuous glucose monitoring; FSF = fractional serum fructosamine; 1;5-AG = 1;5-anhydroglucitol; GCT = glucose challenge test; Combination of OGTT & CGM = Combination of OGTT and CGM; OGTT = oral glucose tolerance test. *: Symptoms of hyperglycaemia and/or weight loss; **: RBG > 11 mmol/L&/HbA1c > 43 mmol/mol (6.1%)/symptoms of hyperglycaemia/weight loss/glycosuria; ***: RBG > 11 mmol/L&/HbA1c > 43 mmol/mol (6.1%)/symptoms of hyperglycaemia/weight loss; ****: One or more of the following criteria: HbA1c ≥ 43 mmol/mol (6.1%), possible diabetic symptoms, FEV1 annual decline > 10%, weight (kg) > 5% annual decline. The top three diagnostic modalities ranked by their superiority indices are presented in Table 2.

Figure 2 displays the four network graphs that enable visualization of the relationship between the examined screening methods and the reference standard.

Network graph A (Figure 2A) summarizes 31 eligible studies and compares all available index tests (*n* = 24) to the reference standard. The most commonly used (19 articles) comparator was HbA1c. The two-day CGM (2d-CGM) (Se: 86%, Sp: 76%), blood capillary sample (BCS) (Se: 70%, Sp: 82%), and three-day CGM (3d-CGM) (Se: 96% Sp: 56%) ranked in the first three positions, according to their SI *(*Table 2*)*.

The network graph B (Figure 2B) represents 25 studies comparing 13 index tests to OGTT 120′. This network was created according to the a priori planned algorithm to avoid potential population overlaps in the first analysis. As in the initial analysis, the majority of articles (17 articles) used HbA1c as the index test. The second most-investigated screening tool was 3d-CGM (six articles). Fructosamine was evaluated in three studies, with fractional serum fructosamine (FSF); 2d-CGM; and 1,5-Anhydroglucitol (1,5-AG) each used in two articles. The glucose challenge test (GCT), glycated albumin, lunch meal, OGTT and CGM combination, combination of different test values, seven-day CGM (7d-CGM), and six-day CGM (6d-CGM) were reported only in single studies. Ranked by SI, 2d-CGM (Se: 87%, Sp: 78%) performed best, mirroring the first analysis. The 2d-CGM was followed by 6d-CGM (Se: 60%, Sp: 77%) and 3d-CGM (Se: 97%, Sp: 54%).

Network graph C (Figure 2C) includes only those index tests that were used in at least two studies. Six different screening tests were investigated. In accordance with the first two analyses, 2d-CGM (Se: 86%, Sp: 78%) was ranked the highest according to SI. The 3d-CGM (Se: 95%, Sp: 53%) was ranked second, and HbA1c (Se: 48%, Sp: 82%) was ranked third.

Network graph D (Figure 2D) was constructed to compare different durations of CGM (between two and seven days) to OGTT. We evaluated data from 10 articles that compared four screening tests. In line with the previous networks, this analysis also ranked 2d-CGM (Se: 88%, Sp: 80%) the highest.

### 3.3. Risk of Bias and Applicability Assessment

The patient selection domain carried a low or unclear risk of bias in the majority of the articles, due to limited reporting in the publications. One study was considered to have a high risk of bias, since the selected patients were known to have abnormal glucose tolerance (but not CFRD), previously diagnosed by OGTT [37]. In the index test domain of QUADAS-2, three records were deemed as having a high risk of bias. In these articles, the authors defined cut-off values for the index tests based on the OGTT results [27,32,40]. The reference standard domain was considered at a low risk of bias in all but one case [41]. In 9% of the papers, the flow and timing domain was considered to have a high risk of bias. The source of bias in this section was the discrepancy between the target and accrued population size in the articles [30,32,41]. All studies had low or unclear applicability concerns in the “patient selection” and “reference standard” domains. The detailed risk of bias and applicability assessment figures are available in Figures S1–S3.

## 4. Discussion

The aim of our study was to robustly compare alternative screening tools to OGTT; therefore, we evaluated currently reported screening methods for CFRD diagnosis for their diagnostic performance. In our results, all four network meta-analyses indicate that the two-day CGM performed best compared to the currently used gold standard: OGTT (T120′).

### 4.1. OGTT

Recent guidelines recommend annual routine OGTT screening for people above the age of 10. Even though OGTT serves as a basis for defining CFRD, its thresholds were established in diabetes without CF, and were not designed to detect hyperglycaemia-associated risk of deterioration in lung function or under-nutrition [39,45]. Considering the practical challenges of OGTT and the low frequency of screening, according to the 2018 report of the Cystic Fibrosis Foundation patient registry (approximately 61.3% of children, aged between 10 and 17, and 33.8% of adults), the need for a more convenient screening method is urgent [3].

### 4.2. HbA1c

HbA1c, the only widely available, simple, relatively cheap diagnostic tool for diabetes, is not recommended for CFRD screening according to recent guidelines, although it is used to guide therapeutic decisions to ensure proper glycaemic control. [4,5]. While an elevated HbA1c ≥ 48 mmol/mol (6.5%) serves as sufficient evidence of hyperglycaemia, a normal HbA1c does not exclude it [5]. Our results were in line with this statement, which is from the International Society for Pediatric and Adolescent Diabetes (ISPAD) Clinical Practice Consensus Guidelines 2018: in our study, HbA1c had a pooled sensitivity of 49% (CI: 35–62%), with a pooled specificity of 81% (CI: 73–87%) (Table S2). Recent studies have suggested lower cut-off values (e.g., 5.4%) for HbA1c to increase sensitivity and specificity [20,46]. In our analysis, HbA1c ranked lower than 2d-CGM in all analysis. We evaluated 17 studies comparing HbA1c to OGTT in Panel B (Table S2), with eight using the recommended 48 mmol/mol (6.5%) cut-off value, and others applying different cut-offs ranging from 5.5 to 6.5 (Table 1).

### 4.3. Glycaemic Biomarkers

Other non-traditional glycaemic biomarkers, such as FSF; fructosamine; 1,5-AG; and glycated albumin are gaining more attention for monitoring short-term glycaemic control in type 1 and type 2 DM, as well as in CFRD [26,27]. The 2010 ADA guideline states that fructosamine has low sensitivity for screening CFRD, while other biomarkers are not mentioned in current guidelines [4,5]. These glycaemic markers ranked in the middle of our ranking table (Table 2). Considering the limited evidence on the diagnostic performance of these markers, further research is necessary to define their cut-off values and exact role in the diagnosis and management of CFRD.

### 4.4. Continuous Glucose Monitoring

The CGM system uses a subcutaneous sensor to measure interstitial fluid glucose levels and provides an average measurement every 5–15 min (depending upon the device). The machine is calibrated by capillary blood glucose levels (usually four times a day). This method has already been used successfully in type 1 and type 2 diabetes patients, as well as in insulin-treated children with CFRD for guiding safe and effective insulin therapy [5,47].

Due to frequent glucose measurements, early glucose abnormalities (both hyper- and reactive hypoglicameia), often preceding CFRD, could be identified by CGM. However, current guidelines do not recommend its use as a screening tool, in spite of its ability to detect elevated glucose levels in CF patients (even when OGTT shows normal values) [22,23,25,29,48], although the clinical significance of intermittent hyperglycaemia detected by CGM is disputed [22,24,29,33]. It is also noteworthy that while OGTT results are not always reproducible and can vary over time, CGM seems to show good reproducibility and reliability [48]. Further advantages of CGM over OGTT include lack of fasting periods, diets, no restrictions on physical activities, and better representation of everyday glucose homeostasis. Our findings demonstrate that from all analysed index tests, CGM (using random glucose levels > 11.1 mmol/L) was relatively the best screening method, as reported in our ranking table (Table 2). In spite of the mildly burdensome calibration process and the substantial required financial investment for the acquisition of a CGM device, in the long term it may help with early diagnosis of glucose abnormalities, allowing earlier treatment and better clinical outcomes for CFRD patients [22,23].

The CGM device is appropriate for both continuous and intermittent measurements. For tracking continuous blood glucose levels, CGM is applied usually for two to seven days. In order to understand the optimal length, we evaluated studies using various lengths of CGM (2–7 days). The diagnostic accuracy seems to be the highest for 2d-CGM. Short measurement length makes the method accessible for a greater proportion with CF, as well as being less inconvenient, facilitating better uptake.

### 4.5. Strength and Limitations

To our knowledge, this is the largest meta-analysis of people with CF evaluating the diagnostic accuracy of the available techniques for the detection of CFRD. Strengths include the selection of homogeneous study populations and standardized OGTT methodology, although the effect of the quality of the studies on the results could not be estimated reliably, since the risk of bias in many studies was unclear.

Limitations include the divergent use of cut-off values of the index tests. Many studies used several different cut-off values for the same index tests. Also, this variability was seen within articles, but in the diagnostic test accuracy (DTA)-NMAs, only one cut-off can be used to avoid overrepresentation of participants. Current guidelines do not state specific cut-off values for CGM or non-traditional glycemic markers. Furthermore, we observed the use of alternative values even where cut-off values are clearly stated in the guidelines (e.g., HbA1c). In order to reduce confounding, we implemented a transparent decision-making algorithm to select the cut-off values used in the analysis.

Originally, we planned to use CGM as a reference standard, as indicated in PROSPERO registration. However, we decided to deviate from our protocol, since OGTT seemed to be the better choice based on methodological recommendations of the Cochrane diagnostic test accuracy (DTA) guidelines, and the professional recommendations of the ADA guideline [6,49].

DTA-NMA allows the use of only one reference standard (as for all studies). This methodology precluded elucidation of whether an index test is better than the reference standard.

## 5. Conclusions

### 5.1. Implication for Practice

Our results indicate that CGM performs well in diagnosing CFRD, which raises the idea of using CGM as an alternative reference standard instead of OGTT in further studies. Two-day CGM seems sufficient, since longer follow-up did not improve global diagnostic performance; this has important financial implications.

### 5.2. Implication for Research

Studies investigating the role of CGM as the gold standard are awaited. In addition, prospective cohort studies should evaluate if abnormalities detected by CGM and missed by OGTT have prognostic value for clinical outcomes, and if patients benefit from treatment initiated based on CGM results. Furthermore, these abnormalities also should be investigated in special risk groups (e.g., severe and less severe genotypes, or pancreatic sufficient and insufficient CF patients).

## Figures and Tables

**Table 1:** Main characteristics of the studies included in the network meta-analysis.

First Author Publ. Year	Country	Number of Centres	Inclusion Time Period	Age Range (Mean)	N^o^ of Participants	Female Ratio (% of Total)	CFRD Prevalence%	Ref. Standard	Index Test	Cut-off Value
Alves et al. 2010 [14]	Brazil	1	August–September 2007	6–16 y	46	35.00	0	OGTT	HbA1c	>48 mmol/mol (6.5%)
Augarten et al. 1999 [15]	Israel	not reported	not reported	13–32 y	14	42.86	0	OGTT	Lundh meal	glucose > 11.1 mmol/L at least once 30–60 min post-test
									HbA1c	>48 mmol/mol (6.5%)
Bismuth et al. 2008 [16]	France	1	1988–2005	(15.0)	206	54.00	18	OGTT	HbA1c	>38 mmol/mol (5.6%)
Boudreau et al. 2016 [17]	Canada	not reported	2004–2015	(25.6)	207	48.30	11	OGTT	HbA1c	>41 mmol/mol (5.9%)
Boudreau et al. 2017 [18]	Canada	not reported	not reported	(35.2)	15	46.67	13	OGTT	7d-CGM	≥11.1 mmol/L
Buck et al. 2000 [19]	Germany	2	not reported	5–33 y	92	42.16	13	OGTT	HbA1c	not reported
Burgess et al. 2016 [20]	United Kingdom	1	March 2009–October 2009	18–61 y (30.0)	94	38.30	6	OGTT	HbA1c	≥43 mmol/mol (6.1%)
									One or more of the following criteria: HbA1c ≥ 43 mmol/mol (6.1%), possible diabetic symptoms, FEV1 annual decline > 10%, weight (kg) > 5% annual decline	
Burgess et al. 2016 valid. [20]	United Kingdom	1	July 2010–July 2012	16–72 y	335	44.20	5	OGTT	HbA1c	>40 mmol/mol (5.80%)
Burgess et al. 2015 [21]	United Kingdom	not reported	June 2013–April 2014	not reported	70	not reported	11	OGTT	BCS 120′	≥11.1 mmol/L
Clemente et al. 2017 [22]	Spain	1	November 2012–May 2015	10–18 y (14.6)	30	53.30	7	OGTT	HbA1c	>40 mmol/mol (5.80%)
									6d-CGM	7.8–11.1 mmol/L + peaks > 11.1 mmol/l > 1% monitoring time
Dobson et al. 2004 [23]	United Kingdom	1	not reported	(27.0)	15	33.33	0	OGTT	2d-CGM	>11.1 mmol/L
Franzese et al. 2008 [24]	Italy	1	not reported	5–20 y	32	68.75	22	OGTT	3d-CGM	>11.1 mmol/L, at any time of the 3d-CGM.
									HbA1c	>48.0 mmol/mol (6.50%)
Jefferies et al. 2005 [25]	Canada	1	December 2002–January 2004	(13.9)	9	63.16	37	OGTT	2d-CGM	>11.1 mmol/L
					19				HbA1c	>48.0 mmol/mol (6.50%)
Kinnaird et al.2010 [26]	United States	1	not reported	19–36 y (26.6)	10	60.00	10	OGTT	HbA1c	>48.0 mmol/mol (6.50%)
									1,5-AG	5.9–33.8 μg/mL
									Fructosamine	290.0 μmol/L
Lam et al. 2018 [27]	Canada	not reported	not reported	20–72 y (34.8)	20	40.00	10	OGTT	FSF	3.7 μmol/g
									Fructosamine	178.97–329.62 μmol/L
Lavie et al. 2015 [28]	Israel	not reported	not reported	(22.8)	55	47.27	7	OGTT	HbA1c	≥48 mmol/mol (6.5%)
Leclercq et al. 2014 [29]	France	1	March 2009–November 2012	(26.6)	58	59.60	0	OGTT	Combination (OGTT or CGM)	OGTT T120′ ≥ 11.1 mmol/L) or CGM ≥ 11.1 mmol/L at least 1x
Lee et al. 2007 [30]	Canada	1	June 2002–May 2003	(32.6)	31	47.37	10	OGTT	GCT T60 ≥ 7.8 mmol/L	
Mainguy et al. 2017 [31]	France	1	June 2009–April 2012	10–17 y (13.1)	29	48.28	10	OGTT	3d-CGM	7.0 mmol/L during the fasting period, or strictly greater than 11.1 mmol/L during the non-fasting period
Martin-Frias et al. 2009 [32]	Spain	not reported	2004–2007	10–22 y	40	not reported	8	OGTT	Combination (OGTT and CGM)	(OGTT T0′ ≥ 6.1 and/or T120′ ≥ 7.8 mmol/L) and (fasting CGM≥ 7.0 mmol/L and/or postprandial CGM ≥ 11.1 mmol/L)
Moreau et al. 2008 [33]	France	1	February 2004–November 2006	(22.3)	49	44.90	20	OGTT	3d-CGM	≥11.1 mmol/L at least once after a meal
O’Riordan et al. 2007 [34]	Ireland	not reported	not reported	children	111	not reported	14	OGTT	2d-CGM	not reported
Schiaffini et al. 2010 [35]	Italy	1	January 2006–December 2006	7.8–18 y (13.3)	17	52.94	6	OGTT	3d-CGM	≥11.1 mmol/L
Schnydera et al. 2016 [36]	Switzerland	1	2002–2015	12–47 y (26.0)	80	48.75	43	OGTT	HbA1c	≥40.0 mmol/mol (5.8%)
Smith et al. 2019 [37]	United Kingdom	not reported	not reported	18–42 y	19	42.00	5	OGTT	HbA1c	not reported
Solomon et al. 2003 [38]	Canada	1	January 1998–January 1999	10–18 y	88	50.00	3	OGTT	HbA1c	>42.0 mmol/mol (6.0%)
Taylor-Cousar et al. 2016 [39]	United States	1	2009–2010	20–65 y (33.8)	18	72.22	6	OGTT	HbA1c	≥41.0 mmol/mol (5.9%)
									3d-CGM	fasting > 7.0 mmol/L, and/or non-fasting > 11.1 mmol/L min2x
Tommerdahl et al. 2019 [40]	United States	1	not reported	10–18 y (14.2)	58	58.62	16	OGTT	HbA1c	>37.0 mmol/mol (5.5%)
									1,5-AG	20.4 mcg/mL
									Fructosamine	225.0 μmol/L
									Glycated albumin	14.0
									FSF	59.0 μmol/g
Widger et al. 2012 [41]	Australia	1	February 2010–June 2011	10–19 y (14.5)	9	72.73	22	OGTT	HbA1c	>48.0 mmol/mol (6.5%)
Winhofer et al. 2019 [42]	Austria	1	September 2012–September 2018	(33.3)	12	33.33	33	OGTT	HbA1c	≥48.0 mmol/mol (6.5%)
Yung et al. 1999 [43]	United Kingdom	1	August l996–May l997	16 y ≤ (27.0)	91	36.26	13	OGTT	HbA1c	>43.0 mmol/mol (6.1%)
									Glycosuria	
									RBG	>11.0 mmol/L
									Fasting glucose (T0′)	>7.7 mmol/L
									Symptoms of hyperglycaemia and/or weight loss	
									RBG > 11.0 mmol/L and/or abnormal HbA1c > 43 mmol/mol (6.1%) and/or the presence of symptoms of hyperglycaemia or unexplained weight loss and/or glycosuria.	
									RBG >11 mmol/L and/or abnormal HbA1c >43 mmol/mol (6.1%) and/or the presence of symptoms of hyperglycaemia or unexplained weight loss.	
									RBG >11.0 mmol/L and/or HbA1c > 43.0 mmol/mol (6.1%)	
Yung et al. 1996 [44]	United Kingdom	not reported	not reported	(25.0)	7	not reported	0	OGTT	T90′	≥11.1 mmol/L
									RVBG: (20′/30′/40′/50′/60′/75′/90′)	≥11.1 mmol/L

Participant age is range or mean. Abbreviations: y = years; d = days; OGTT = oral glucose tolerance test; BCS = blood capillary sample; CGM = continuous glucose monitoring; HbA1c = haemoglobin A1c; 1,5-AG = 1,5-anhydroglucitol; GCT = glucose challenge test (50 g glucose load administered in the non-fasting state and followed by glucose measurement 1-h later.); RBG = random blood glucose; RVBG = random venous blood glucose.

**Table 2:** Ranking table of the top three index tests by superiority indices.

Ranking of Index Test	Index Test	SI Mean (95%CI)	Pooled Sensitivity Mean (95% CI)	Pooled Specificity Mean (95% CI)
*Network A*				
#1	2 day-CGM	18.56 (0.26–43.00)	86% (40–100%)	76% (17–90%)
#2	BCS 120′	17.52 (0.0–43.00)	70% (19–99%)	82% (21–100%)
#3	3 day-CGM	9.30 (0.54–25.00)	96% (74–100%)	56% (8–62%)
*Network B*				
#1	2 day-CGM	12.66 (0.33–25.00)	87% (43–100%)	78% (37–98%)
#2	6 day-CGM	7.03 (0.06–21.00)	60% (10–97%)	77% (27–100%)
#3	3 day-CGM	5.68 (0.33–15.00)	97% (82–100%)	54% (35–71%)
*Network C*				
#1	2 day-CGM	5.58 (0.33–11.00)	86% (43–100%)	78% (36–98%)
#2	3 day-CGM	1.67 (0.33–5.00)	95% (72–95%)	53% (36–69%)
#3	HbA1c	1.61 (0.14–7.00)	48% (35–62%)	82% (75–86%)
*Network D*				
#1	2 day-CGM	3.44 (0.33–7.00)	88% (47–100%)	80% (42–98%)
#2	3 day-CGM	2.40 (0.33–5.00)	96% (76–95%)	59% (46–70%)
#3	6 day-CGM	1.20 (0.14–5.00)	55% (6–100%)	79% (31–100%)

The top three diagnostic modalities ranked by their superiority indices in all four analysis. Network A represents the first analysis, which included all eligible studies (*n* = 31) and compared 24 index tests to the reference standard. Two day-CGM, BCS 120′, and 3 day-CGM ranked in the first three positions, according to the SI. A more detailed ranking table is available for all analysis in Table S3. Network B lists the ranking of the second analysis, which included 25 articles and 13 index tests. Among these 13 index tests, 2 day-CGM, 6 day-CGM, and 3 day-CGM ranked the highest. A more detailed ranking table is available in the Table S4. Network C shows the results of the third analysis, comparing those index tests that were used in at least two different articles to the reference standard. From 23 articles, 6 different screening methods were evaluated. In the first three positions, 2 day-CGM, 3 day-CGM, and HbA1c were ranked. A more detailed ranking table is available in Table S5. Network D demonstrates the ranking of different lengths of CGM. Two day-CGM seems to be relatively the best diagnostic method, while 3 day-CGM and 6 day-CGM took second and third places. The full table can be seen in Table S6. In all four analyses, according to the SI, 2 d-CGM ranked the highest. Abbreviations: SI: superiority index, CI: 95% confidence interval, BCS: blood capillary sample, CGM: continuous glucose monitoring, HbA1c: haemoglobin A1c.

## Data Availability

Not applicable.

## Supplementary Materials

The following are available online at https://www.mdpi.com/article/10.3390/biom11040520/s1, Figure S1: Risk of bias assessment Figure S2: Risk of bias assessment, applicability concerns. Figure S3: Bar graphs of QUADAS-2 risk of bias and applicability assessment Table S1: Detailed search strategy. Table S2: 2 × 2 contingency tables with true positive (TP), true negative (TN), false positive (FP) and false-negative (FN) values of all index tests from all eligible studies. Table S3: First analysis index tests ranked by their superiority indexes. Table S4: Second analysis which included 25 articles and ranked 13 index tests by their superiority indices. Table S5: Index tests used in at least two articles, ranked by their superiority indices. Table S6: Different length CGMs compared by their superiority indices.
