# Supplementary material for: Screening Methods for Diagnosing Cystic Fibrosis-Related Diabetes: A Network Meta-Analysis of Diagnostic Accuracy Studies
Source: Biomolecules. 2021 Mar 31;11(4):520. doi: 10.3390/biom11040520 (PMC8065857; doi:10.3390/biom11040520)
Supplement: Supplementary file 1 [file biomolecules-11-00520-s001.pdf]

Online Supplemental Materials

Screening methods for diagnosing cystic fibrosis - related diabetes: A network meta-analysis of diagnostic accuracy studies

Short title: Diagnostic methods for CFRD screening

Vera Dóra Izsák [1,2,3], Alexandra Soós [1], Zsolt Szakács [1], Péter Hegyi [1,4] Márk Félix Juhász [1], Orsolya Varannai [1,2,3], Ágnes Rita Martonosi [1,2,3], Mária Földi [1,4], Alexandra Kozma [1], Zsolt Vajda[2,], James AM Shaw[5], Andrea Párniczky [1,2,3]

1. Institute for Translational Medicine, Medical School, University of Pécs, Pécs, 7624, Hungary

2. Heim Pál National Pediatrics Institute, Budapest,1089, Hungary

3. Doctoral School of Clinical Medicine, University of Szeged, Szeged, 6720, Hungary

4. János Szentágothai Research Center, University of Pécs, Pécs, 7624, Hungary

5. Translational and Clinical Research Institute, Newcastle University, Newcastle upon Tyne, NE2 4HH, UK;

Corresponding author:

Andrea Párniczky M.D., Ph.D.

Telephone: 0036-0672-536-246

E-mail: andrea.parniczky@gmail.com

**Database**	**Date of search**	**Search terms**	**Selection options**	**Number of records**
MEDLINE	Beginning of October 9 2019	(„cystic fibrosis” OR cftr OR mucoviscidosis) AND (diabet* OR prediabet* OR „impaired glucose tolerance” OR IGT OR „impaired fasting glucose” OR IFG OR indeterminate glucose tolerance OR INDET)	None used	1934 records
Embase		('cystic fibrosis':ti,ab,kw OR cftr:ti,ab,kw OR mucoviscidosis:ti,ab,kw) AND (diabet*:ti,ab,kw OR prediabet*:ti,ab,kw OR 'impaired glucose tolerance':ti,ab,kw OR igt:ti,ab,kw OR 'impaired fasting glucose':ti,ab,kw OR ifg:ti,ab,kw OR 'indeterminate glucose tolerance':ti,ab,kw OR indet:ti,ab,kw)--		3177 records
Scopus		TITLE-ABS-KEY (("cystic fibrosis" OR cftr OR mucoviscidosis) AND (diabet* OR prediabet* OR "impaired glucose tolerance" OR igt OR "impaired fasting glucose" OR ifg OR "indeterminate glucose tolerance" OR indet))	None used	3510 records

CENTRAL		(„cystic fibrosis” OR cftr OR mucoviscidosis) AND (diabet* OR prediabet* OR „impaired glucose tolerance” OR IGT OR „impaired fasting glucose” OR IFG OR indeterminate glucose tolerance OR INDET)		253 records<br>- review 83,<br>- protocol 12<br>- **trials 157**
Web of science		TS=("cystic fibrosis" OR cftr OR mucoviscidosis) AND TS=(diabet* OR prediabet* OR "impaired glucose tolerance" OR IGT OR "impaired fasting glucose" OR IFG OR "indeterminate glucose tolerance" OR INDET)		2062 records

Supplementary Table 1: Detailed search strategy. The following search key was developed and adapted for each database without any restrictions: „cystic fibrosis” OR „cftr” OR „mucoviscidosis”; AND „diabet* OR „prediabet* OR „impaired glucose tolerance” OR „IGT” OR „impaired fasting glucose” OR „IFG” OR „indeterminate glucose tolerance” OR „INDET”.

**First Author**	**Exclusion criteria**	**Reference standard**	**Index tests**	**TP**	**FP**	**FN**	**TN**
Alves et al. 2010	acute exacerbations within a month, steroid or antibiotic use	OGTT 120'	HbA1c	0	0	0	46
Augarten et al. 1999	not reported	OGTT 120'	Lundah meal	0	6	0	8
			HbA1c	0	4	0	10
Bismuth et al.2008	acute exacerbations, steroid or antibiotic use	OGTT 120'	HbA1c	33	33	4	136
Boudreau et al. 2016	not reported	OGTT 120'	HbA1c	13	50	9	135
Boudreau et al. 2017	not reported	OGTT 120'	7d-CGM	2	8	0	5
Buck et al. 2000	steroid use, pregnancy	OGTT 120'	HbA1c	3	8	10	81
Burgess et al. 2016		OGTT 120'	HbA1c	3	32	3	56

	known CFRD, transplant recipients, acute exacerbation		One or more of the following criteria: HbA1c ≥ 6.1% , Possible diabetic symptoms, FEV1 annual decline >10%, Weight (kg) > 5% annual decline.	4	38	2	50
Burgess et al. 2016 valid	known CFRD, transplant recipients, acute exacerbation	OGTT 120'	HbA1c	15	150	1	169
Burgess et al. 2015	not reported	OGTT 120'	BCS 120'	7	0	1	62
Clemente et al. 2017	age < 10years or > 18years<br>non-genetically confirmed CF, acute exacerbations within 6 weeks, steroid or growth hormone use, insulin therapy, transplant recipients	OGTT 120'	6d-CGM	1	0	1	28
			HbA1c	0	0	2	28
Dobson et al. 2004	age <10 years, known CFRD, current medication, or condition that would interfere with glucose tolerance	OGTT 120'	2d-CGM	0	10	0	5
Franzese et al. 2008		OGTT 120'	3d-CGM	7	13	0	12

	acute pulm. exacerbations, steroid or antibiotic use in the previous 4 weeks		HbA1c	1	2	6	23
Jefferies et al. 2005	pancreatic sufficiency, acutely severely unwell, steroid or growth hormone use	OGTT 120'	2d-CGM	4	0	0	5
			HbA1c	5	0	2	12
Kinnaird et al. 2010	age < 18 years, known CFRD, chronic kidney disease, renal tubular defects, pregnancy, end stage liver disease with cirrhosis,	OGTT 120'	HbA1c	0	0	1	9
			1,5-AG	0	0	1	9
			Fructosamine	0	0	1	9
Lam et al. 2018	non-clinically stable adult patients, known CFRD, transplant recipients, pregnancy	OGTT 120'	FSF	2	8	0	10
			Fructosamine	0	1	2	17
Lavie et al. 2015	age <10 years, known CFRD, acute exacerbation within 6 weeks, steroid use, cirrhosis, pregnancy	OGTT 120'	HbA1c	0	7	4	44
Leclercq et al. 2014	known CFRD, steroid use, liver disease, transplant recipients,	OGTT 120'	Combination (OGTT or CGM(11,1mmol/L))	5	12	0	35
Lee et al. 2007	known CFRD, transplant recipients	OGTT 120'	GCT	3	17	0	11

Mainguy et al. 2017	age < 10 years >18 years, non-genetically confirmed CF patients, pancreatic sufficiency, acute. exacerbation within 4 weeks, FEV1<40%, steroid use, previous glucose abnormalities; CGMS monitoring difficulties, transplant (awaiting) recipients, parenteral nutrition, participation in other study	OGTT 120'	3d-CGM	3	8	0	18
Martin-Frias et al. 2009	steroid use, any underlying illness	OGTT 120'	Combination (OGTT and CGM(11,1mmol/L))	1	6	11	22
Moreau et al. 2008	CF subjects with fasting glycemia> 7.0 mmol/L age < 15 years, steroid use acute exacerbation or infection that would interfere with glucose tolerance, previous glucose abnormalities	OGTT 120'	3d-CGM	10	17	0	22
O'Riordan et al. 2007	not reported	OGTT120'	2d-CGM	16	13	0	82
Schiaffini et al. 2010	>18 years, acute exacerbation or infection, steroid use within 3 months	OGTT 120'	3d-CGM	1	5	0	11

Schnydera et al. 2016	non genetically confirmed CF, FEV1>45%	OGTT 120'	HbA1c	29	33	5	13
Smith et al. 2019	not reported	OGTT 120'	HbA1c	1	1	0	17
Solomon et al. 2003	known CFRD, clinical CFRD developed during the study period acute. exacerbations, steroid or antibiotic use, pregnancy,	OGTT 120'	HbA1c	1	16	2	69
Taylor-Cousar et al 2016	not reported	OGTT 120'	HbA1c	0	0	1	17
			3d-CGM	1	4	0	13
Tommerdahl et al. 2019	known CFRD, steroid use, within 3 months, insulin or other medications that would interfere with glucose tolerance, hospitalizations within 6 weeks, pregnancy	OGTT 120'	HbA1c	7	29	2	20
			1,5-AG	6	18	3	31
			Fructosamine	9	32	0	27
			Glycated albumin	2	5	7	44
			FSF	4	8	5	41
Widger et al.2012	known CFRD, pancreatic sufficiency, acute exacerbation, steroid use within 6 weeks,	OGTT 120'	HbA1c	1	0	1	7

Winhofer et al. 2019	known CFRD, non-lung transplanted CF patients	OGTT 120'	HbA1c	1	0	3	8
Yung et al. 1999	acute exacerbation with antibiotic therapy within 6 weeks, steroid use, enteral feeding, pregnancy	OGTT 120'	HbA1c	10	9	2	70
			glycosuria	2	2	10	77
			Random blood glucose (RBG)	4	2	8	77
			Fasting glucose(T0’)	3	0	9	79
			symptoms of hyperglycaemia and/or weight loss	7	10	5	69
			RBG (>11.0) and/or abnormal HbA1c (>6.1) and/or the presence of symptoms of hyperglycaemia or unexplained weight loss and/or glycosuria.	11	17	1	62
			RBG (>11) and/or abnormal HbA1c (>6.1) and/or the presence	11	17	1	62

			of symptoms of hyperglycaemia or unexplained weight loss.				
			RBG (>11) and/or HbA1c (>6.1)	10	11	2	68
Yung et al. 1996	non- clinically stable patients, known CFRD	OGTT 120'	T90	0	1	0	6
			RVBG:(20'/30'/40'/50'/60'/75'/90')	0	4	0	3

Supplementary Table 2: 2x2 contingency tables with true positive (TP), true negative (TN), false positive (FP) and false-negative (FN) values of all index tests from all eligible studies.

Abbreviations: d:days OGTT: oral glucose tolerance test, BCS: blood capillary sample, CGM: continuous glucose monitoring, HbA1c: haemoglobin A1c, FSF: fractional serum fructosamine, 1,5-AG: 1,5 - anhydroglucitol, GCT: glucose challenge test (50-g glucose load administered in the non-fasting state and followed by glucose measurement 1-h later.), RBG: random blood glucose, RVBG: random venous blood glucose

**Rank**	**Index test**	**Superiority index mean (CI)**	**Pooled sensitivity mean (CI)**	**Pooled specificity mean (CI)**
#1	2day-CGM	18.56 (0.26-43.0)	0.86 (0.4-1.0)	0.76 (0.17-0.9)
#2	BCS 120’	17.52 (0.0-43.0)	0.70 (0.19-0.99)	0.82 (0.21-1)
#3	3day-CGM	9.30 (0.54-25.0)	0.96 (0.74-1.0)	0.56 (0.08-0.62)
#4	6day-CGM	7.95 (0.03-39)	0.5 (0.06-0.93)	0.76 (0.23-0.98)
#5	Combination of OGTT or CGM	6.48 (0.04-33)	0.77 (0.3-1.0)	0.6 (0.19-0.75)
#6	RBG (>11mmol/L) and/or HbA1c (>6.1%)	5.62 (0.03-33)	0.63 (0.17-0.96)	0.68 (0.2-0.84)
#7	RVBG T90’	5.58 (0.03-39)	0.5 (0.0-1.0)	0.69 (0.23-0.89)
#8	**	5.52 (0.04-31.05)	0.68(0.22-0.98)	0.67(0.19-0.8)
#9	Fasting glucose	5.46 (0.03-27.0)	0.32 (0.04-0.77)	0.85(0.2-1.0)
#10	***	5.43 (0.03-33.0)	0.69 (0.21-0.99)	0.62 (0.21-0.79)
#11	GCT	4.23 (0.03-33.0)	(0.78(0.27-1)	0.47 (0.23-0.64)

#12	RBG	4.03 (0.03-29.0)	0.39(0.06-0.82)	0.77 (0.22-0.96)
#13	7day CGM	3.58 (0.03-27.0)	0.75(0.27-1.0)	0.46 (0.22-0.62)
#14	Lundh meal	3.20 (0.02-29)	0.5 (0-1.0)	0.57 (0.21-0.72)
#15	symptoms of hypreglicaemia and/or weight loss	3.03 (0.03-27.0)	0.48 (0.1-0.89)	0.68 (0.21-0.85)
#16	Glycated albumin	2.9 (0.03-25.0)	0.3 (0.03-0.77)	0.77 (0.2-0.94)
#17	FSF	2.88 (0.03-25)	0.57 (0.19-0.91)	0.65 (0.18-0.79)
#18	Fructosamine	2.74 (0.04-23)	0.46 (0.14-0.81)	0.75 (0.15-0.86)
#19	HbA1c	2.69 (0.47-15)	0.54 (0.41-0.66)	0.8 (0.03-0.82)
#20	1,5-AG	2.47 (0.03-23)	0.41 (0.08-0.81)	0.75 (0.17-0.87)
#21	One or more of the following criteria: HbA1c ≥ 6.1% , Possible diabetic symptoms, FEV1 annual decline >10%, Weight (kg) > 5% annual decline.	2.35 (0.03-23)	0.57(0.15-0.95)	0.59 (0.21-0.75)
#22	glycosuria	2.18 (0.02-19.0)	0.28 (0.2-0.76)	0.74 (0.22-0.94)
#23	RVBG (20'/30'/40'/50'/60'/75'/90')	1.94 (0.02-21.0)	0.52 (0.0-1.0)	0.46 (0.23-0.63)
#24	Combination of OGTT and CGM	0.96 (0.02-7.0)	0.27 (0.01-0.79)	0.66 (0.21-0.82)

38 **Supplementary Table 3**: First analysis index tests ranked by their superiority indexes

Network A represents the first analysis, which included all eligible studies (n=31) and compared 24 index tests to the reference standard by superiority indices. 2day-CGM, BCS120' and 3day-CGM ranked in the first three positions according to SIs.

Abbreviations: OGTT: oral glucose tolerance test, BCS: blood capillary sample, CGM: continuous glucose monitoring, HbA1c: haemoglobin A1c, FSF: fractional serum fructosamine, 1,5-AG: 1,5 - anhydroglucitol, GCT: glucose challenge test (50-g glucose load administered in the non-fasting state and followed by glucose measurement 1-h later.), RBG: random blood glucose, RVBG: random venous blood glucose, *:Symp. of hyperglycaemia and/or weight loss **: RBG>11mmol/L&/HbA1c >6.1%&/symptoms of hyperglycaemia/weight loss &/glycosuria, ***: RBG>11mmol/L&/HbA1c >6.1%&/symptoms of hyperglycaemia / weight loss, ****: One or more of the following criteria: HbA1c ≥ 6.1% , Possible diabetic symptoms, FEV1 annual decline >10%, Weight (kg) > 5% annual decline.

**Ranking of Graph**	**Index test**	**Superiority index mean (CI)**	**Pooled sensitivity mean (CI)**	**Pooled specificity mean (CI)**
**#1**	2day-CGM	12.66 (0.33-25)	0.87 (0.43-1.0)	0.78 (0.37-0.98)
#2	6day-CGM	7.03 (0.06-21)	0.6 (0.1-0.97)	0.77 (0.27-1.0)
#3	3day-CGM	5.68 (0.33-15)	0.97 (0.82-1.0)	0.54 (0.35-0.71)
#4	FSF	2.60 (0.07-15)	0.54 (0.18-0.9)	0.69 (0.29-0.94)
#5	HbA1c	2.58 (0.27-11)	0.49 (0.36-0.62)	0.81 (0.73-0.87)

#6	GCT	2.54 (0.05-17)	0.75 (0.26-1.0)	0.46 (0.07-0.88)
#7	Fructosamine	2.48 (0.33-15)	0.45 (0.14-0.79)	0.76 (0.45-0.95)
#8	1,5-AG	2.47 (0.06-15)	0.43 (0.09-0.81)	0.75 (0.37-0.96)
#9	Glycated albumin	2.40 (0.04-15)	0.30 (0.03-0.77)	0.78 (0.30-0.99)
#10	7 day-CGM	2.29 (0.05-15)	0.76 (0.25-1,0)	0.45 (0.09-0.88)
#11	Lundh meal	2.08 (0.04-15)	0.51 (0-1.0)	0.57 (0.16-0.92)
#12	One or more of the following criteria: HbA1c ≥ 6.1% , Possible diabetic symptoms, FEV1 annual decline >10%, Weight (kg) > 5% annual decline.	1.75 (0.05-13)	0.55 (0.13-0.93)	0.6 (0.18-0.92)
#13	Combination (OGTT and CGM (11.1)	0.93 (0.04-9)	0.3 (0.00-0.79)	0.62 (0.16-0.96)

Supplementary Table 4.: Second analysis which included 25 articles and ranked 13 index tests by their superiority indices.

Network B assessed studies after excluded those, which raised potential risk for overlapping populations. From 25 articles among 13 index tests, 2day-CGM, 6day-CGM and 3day-CGM ranked the highest.

Abbreviations: OGTT: oral glucose tolerance test, CGM: continuous glucose monitoring, HbA1c: haemoglobin A1c, FSF: fractional serum fructosamine, 1,5-AG: 1,5 - anhydroglucitol, GCT: glucose challenge test (50-g glucose load administered in the non-fasting state and followed by glucose measurement 1-h later.)

Rank	Index test	Superiority index mean (CI)	Pooled sensitivity mean (CI)	Pooled specificity mean (CI)
**#1**	2day-CGM	5,58 (0,33-11,0)	0,86 (0,43-1,00)	0,78 (0,36-0,98)
#2	3day-CGM	1,67 (0,33-5)	0,95 (0,72-0,95)	0,53 (0,36-0,69)
#3	HbA1c	1,61 (0,14-7)	0,48 (0,35-0,62)	0,82 (0,75-0,86)
#4	Fructosamine	1,44 (0,11-7)	0,45 (0,13-0,79)	0,77 (0,42-0,95)
#5	FSF	1,39 (0,11-7)	0,54 (0,17-0,89)	0,69 (0,31-0,94)
#6	1,5-AG	1,30 (0,09-7)	0,41 (0,09-0,8)	0,76 (0,36-0,97)

Supplementary Table 5. : Index tests used in at least two articles, ranked by their superiority indices.

From 23 articles 6 different screening methods were evaluated. In the first three positions 2day-CGM, 3day-CGM, and HbA1c were ranked.

Abbreviations: CGM: continuous glucose monitoring, HbA1c: haemoglobin A1c, FSF: fractional serum fructosamine, 1,5-AG: 1,5 - anhydroglucitol,

Ranking of graph D	Index test	Superiority index mean (CI)	Pooled sensitivity mean (CI)	Pooled specificity mean (CI)
**#1**	2day-CGM	3.44 (0.33-7)	0.88 (0.47-1.00)	0.80 (0.42-0.98)
#2	3day-CGM	2.40 (0.33-5)	0.96 (0.76-0.95)	0.59 (0.46-0.70)
#3	6day-CGM	1.20 (0.14-5)	0.55 (0.06-1.00)	0.79 (0.31-1.00)
#4	7day-CGM	0.62 (0.14-3)	0.75 (0.24-1.00)	0.41 (0.07-0.86)

Supplementary Table 6. Different length CGMs compared by their superiority indices

Network D demonstrate the ranking of different length of CGMs by their superiority indices. 2day-CGM seems to be relatively the best diagnostic method, while 3day-CGM and 6day-CGM took second and third places.

Abbreviations: CGM: continuous glucose monitoring

1.1 Risk of bias and applicability assessment

The patient selection domain carried a low or unclear risk of bias in the majority of the articles due to limited reporting in the publications. One record was considered to have a high risk of bias since the selected patients were known to have some kind of glucose abnormality (not CFRD) previously diagnosed by OGTT [37]. In the index test domain of QUADAS-2, 3 records were deemed as high risk of bias. In these articles they defined cut-off values for the index tests based on the OGTT results [27, 32, 40]. The reference standard domain was considered low risk of bias in all but one case [41]. In 9% of the papers the flow and timing domain was considered high risk of bias. The source of bias in this section was the discrepancy between the target and final population size in the articles [30, 32, 41]. All studies had low or unclear applicability concerns in the 'Patient Selection' and 'Reference Standard' domains. The detailed risk of bias and applicability assessment figures are available in *Supplementary Figure 1, 2 and 3.*

High | Unclear | Low

Risk of Bias

	Patient Selection	Index Test: Fasting glucose>7,7mmol/l	Index Test: Comb.(OGTT and CGM(11,1)	Index Test: Presence of glycosuria	Index Test: RBG>11mmol/l	Index Test: *	Index Test: **	Index Test: HbA1c	Index Test: ***	Index Test: RBG>11mmol/l&/HbA1c>6.1%	Index Test: RBG:T(20/30/40/50/60/75/90)	Index Test: Fructosamine	Index Test: 1,5-anhydroglucitol(1,5-AG)	Index Test: Glycated albumin	Index Test: FSF	Index Test: 3d-CGM	Index Test: 2d-CGM	Index Test: Combination(OGTT and CGM(11,1)	Index Test: GCT	Index Test: 6d-CGM	Index Test: BCS 120'	Index Test: ****	Index Test: Lundah meal	Index Test: 7d-CGM	Reference Standard	Flow and Timing
Alves 2010	?							?																	+	+
Augarten 1999	?							?															+		+	?
Bismuth 2008	?							+																	+	?
Boudreau 2016	?							+																	+	+
Boudreau 2017	?																							+	+	+
Buck 2000	?							?																	+	+
Burgess 2015	?																				+				+	+
Burgess 2016 (1)	+							+														+			+	?
Burgess et al. 2016(2)	+							+																	+	+
Clemente 2017	+							+												+					+	+
Dobson 2004	?																+								+	?
Franzese 2008	?							+								+									+	?
Jefferies 2005	?							+								+									+	?
Kinnaird 2010	+							+				+	+												+	+
Lam 2018	+											−			−										+	+
Lavie 2015	+							+																	+	+
Leclercq 2014	?															+		+							+	?
Lee 2007	+																		+						+	−
Mainguy 2017	+															+									+	+
Martin-Frias 2009	?																	−							+	−
Moreau 2008	+															+									+	+
O'Riordan 2007	?																?								+	?
Schiaffini 2010	?															+									+	?
Schnydera 2016	?							+																	+	?
Smith 2019	−							?																	+	+
Solomon 2003	?							+																	+	?
Taylor-Cousar 2016	?							+								+									+	?
Tommerdahl 2019	+							−				−	−	−	−										+	+
Widger 2012	?							+																	?	−
Winhofer 2019	?							+																	+	+
Yung 1996	?		+								+														+	+
Yung 1999	?	+		+	+	+	+	+	+	+															+	+

Supplementary Figure 1: Risk of bias assessment *:Symp. of hyperglycaemia and/or weight loss**: RBG>11mmol/L&/HbA1c >6.1%&/symptoms of hyperglycaemia/weight loss &/glycosuria, ***:RBG>11mmol/L&/HbA1c >6.1%&/symptoms of hyperglycaemia/weight loss, ****: One or more of the following criteria: HbA1c ≥ 6.1% , Possible diabetic symptoms, FEV1 annual decline >10%, Weight (kg) > 5% annual decline.

Applicability Concerns

	Patient Selection	Index Test: Fasting glucose>7,7mmol/l	Index Test: T90' Blood glucose	Index Test: Presence of glycosuria	Index Test: RBG>11mmol/l	Index Test: *	Index Test: **	Index Test: HbA1c	Index Test: ***	Index Test: RBG>11mmol/l&/HbA1c>6.1%	Index Test: RBG:T(20/30/40/50/60/75/90)	Index Test: Fructosamine	Index Test: 1,5-anhydroglucitol(1,5-AG)	Index Test: Glycated albumin	Index Test: FSF	Index Test: 3d-CGM	Index Test: 2d-CGM	Index Test: Combination(OGTT and CGM(11,1)	Index Test: GCT	Index Test: 6d-CGM	Index Test: BCS 120'	Index Test: ****	Index Test: Lundah meal	Index Test: 7d-CGM	Reference Standard
Alves 2010	+							+																	+
Augarten 1999	?							?															+		+
Bismuth 2008	?							+																	+
Boudreau 2016	+							+																	+
Boudreau 2017	?																							+	+
Buck 2000	?							+																	+
Burgess 2015	?																				+				+
Burgess 2016 (1)	+							+														+			+
Burgess et al. 2016(2)	+							+																	+
Clemente 2017	+							+												+					+
Dobson 2004	?																+								+
Franzese 2008	?							+								+									+
Jefferies 2005	?							+								+									+
Kinnaird 2010	+							+				+	+												+
Lam 2018	+											−			−										+
Lavie 2015	+							+																	+
Leclercq 2014	?															+		+							+
Lee 2007	+																		+						+
Mainguy 2017	+															+									+
Martin-Frias 2009	?																	−							+
Moreau 2008	+															+									+
O'Riordan 2007	?																?								+
Schiaffini 2010	?															+									+
Schnydera 2016	+							+																	+
Smith 2019	?							?																	+
Solomon 2003	+							+																	+
Taylor-Cousar 2016	?							+								+									+
Tommerdahl 2019	+							−				−	−	−	−										+
Widger 2012	?							?																	?
Winhofer 2019	?							+																	+
Yung 1996	?		+								+														+
Yung 1999	+	+		+	+	+	+	+	+	+															+

Supplementary Figure 2: Risk of bias assessment, applicability concerns *:Symp. of hyperglicaemia and/or weight loss**:RBG>11mmol/L&/HbA1c >6.1%&/symptoms of hyperglycaemia/weight loss &/glycosuria, ***:RBG>11mmol/L&/HbA1c >6.1%&/symptoms of hyperglycaemia/weight loss, ****: One or more of the following criteria: HbA1c ≥ 6.1% , Possible diabetic symptoms, FEV1 annual decline >10%, Weight (kg) > 5% annual decline.

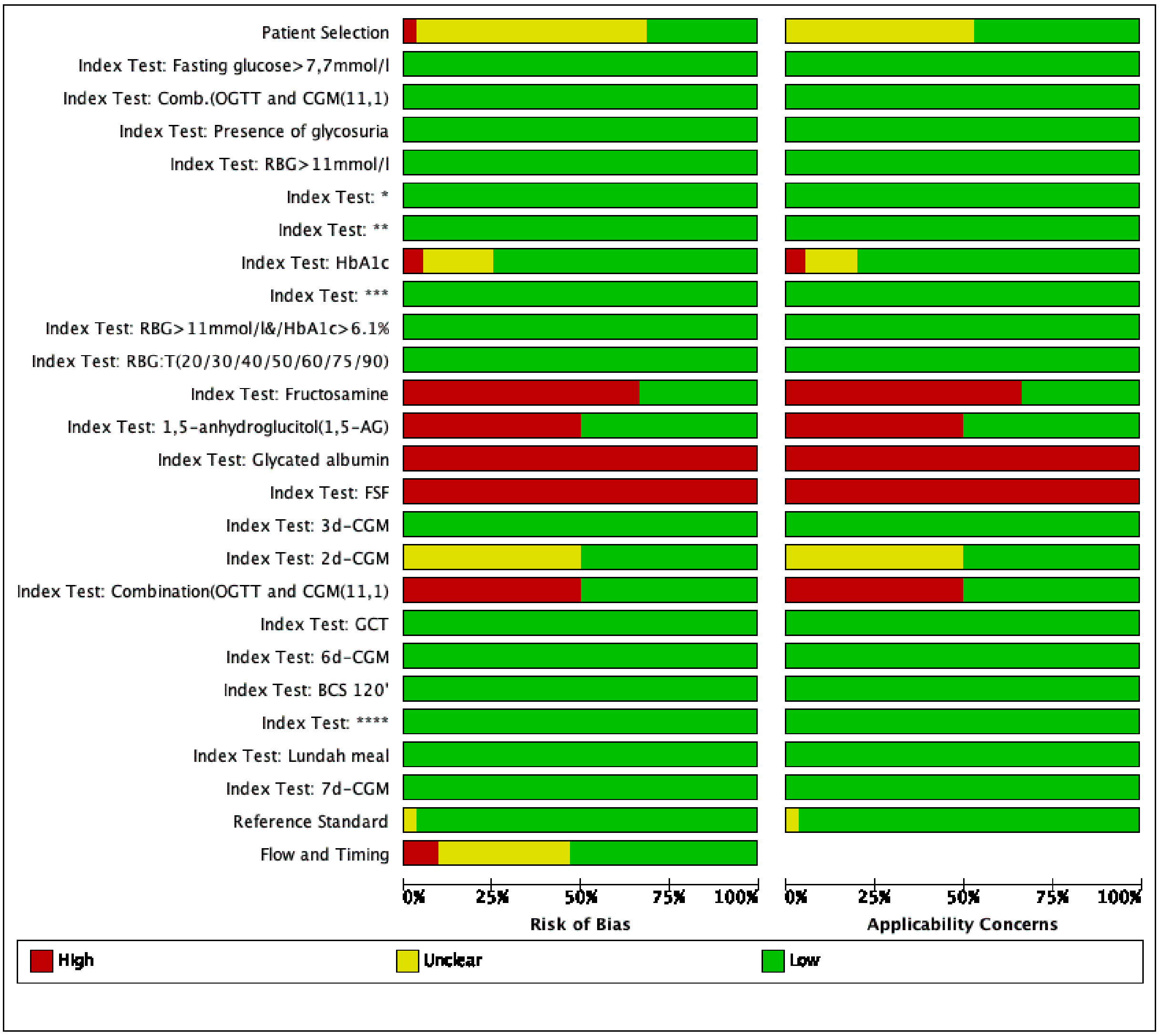

Supplementary Figure 3. Bar graphs of QUADAS-2 risk of bias and applicability assessment d:days, CGM: continuous glucose monitoring, FSF: fractional serum fructosamine, 1,5-AG: 1,5- anhydroglucitol, GCT: glucose challenge test, Comb.OGTT & CGM: Combination of OGTT and CGM, OGTT: oral glucose tolerance test, *:Symp. of hyperglycaemia and/or weight loss **: RBG>11mmol/L&/HbA1c >6.1%&/symptoms of hyperglycaemia/weight loss

&/ glycosuria, ***: RBG>11mmol/L&/HbA1c >6.1%&/symptoms of hyperglycaemia/weight loss, ****: One or more of the following criteria: HbA1c ≥ 6.1% , Possible diabetic symptoms, FEV1 annual decline >10%, Weight (kg) > 5% annual decline.
